# Postoperative Altered Mental Status: A Case Report and Diagnostic Dilemma

**DOI:** 10.7759/cureus.68368

**Published:** 2024-09-01

**Authors:** Nicole Ann E Villa, Dragos G Pausescu, Eduardo D Espiridion

**Affiliations:** 1 Psychiatry, Drexel University College of Medicine, Philadelphia, USA; 2 Psychiatry, Reading Hospital Tower Health Systems, West Reading, USA; 3 Psychiatry, West Virginia School of Osteopathic Medicine, Lewisburg, USA; 4 Psychiatry, Philadelphia College of Osteopathic Medicine, Philadelphia, USA

**Keywords:** cognitive disturbances, postoperative delirium, surgical complications, diagnostic evaluation, altered mental status

## Abstract

Postoperative altered mental status (AMS) is a prevalent and complex issue that poses a significant diagnostic challenge in the clinical setting. Clinical presentation consists of cognitive disturbances that can range from mild confusion to coma. Given the complexity and variability of AMS, each patient requires a careful and thorough evaluation to identify the underlying cause. Thus, we present a case of a 57-year-old male with confirmed left knee septic arthritis, whose admission was complicated by AMS following several incision and drainage procedures. We highlight the importance of considering a broad differential diagnosis when evaluating postoperative AMS, including electrolyte disturbances and systemic conditions. The case also discusses the importance of early recognition, interdisciplinary collaboration, and a comprehensive diagnostic strategy. By adopting a comprehensive and collaborative approach, healthcare providers can optimize patient outcomes and minimize complications in postoperative AMS cases. The patient's AMS was ultimately attributed to a combination of metabolic disturbances, drug-related factors, and systemic conditions. His severe psychiatric symptoms were successfully managed with targeted pharmacologic interventions.

## Introduction

Postoperative altered mental status (AMS) is a condition where patients experience cognitive disturbances after surgery, ranging from mild confusion to coma. This condition affects a significant number of surgical patients. AMS can manifest as a spectrum of cognitive disturbances, with an incidence of 35% to 73% of surgical patients having postoperative AMS, depending on factors such as age, comorbidities, and the type of surgery performed [1-5]. 

Patients with AMS may present with many symptoms, including confusion, disorientation, agitation, hallucinations, and fluctuations in consciousness. The onset of these symptoms varies and can occur immediately after surgery or up to several days postoperatively. This variability in presentation complicates the diagnosis of postoperative AMS [3]. When postoperative AMS is suspected, etiologies such as anesthesia-related factors, surgical stress, preexisting medical conditions, and medication effects need to be considered as many causes of AMS cases are reversible [6,7]. Diagnosis and appropriate management of AMS are needed to optimize patient outcomes as delayed or missed diagnoses can lead to prolonged hospitalization, increased morbidity, and higher healthcare costs [2,3]. Understanding the causes of AMS is crucial for timely diagnosis and effective management. 

This case report will explore the clinical presentation, diagnostic evaluation, and management of postoperative AMS through a comprehensive review of potential differential diagnoses that should be considered for these patients. This report will help to provide a better understanding of the various etiologies for postoperative AMS, which may provide insights into effective diagnostic and treatment strategies. Thus, we present a case of a 57-year-old male with suspected septic arthritis, whose admission was complicated by AMS following several incision and drainage procedures.

## Case presentation

The patient, a 57-year-old male with a history of depression and anxiety, presented with severe left knee pain and swelling. The patient notes that he missed a step and hit his knee against the stairwell about two days prior to the presentation. He had since been having increasing pain, edema, and erythema with purulent drainage from a previous laceration on the anterior portion of his left knee. He mentions that one month prior to the presentation, he was admitted by trauma and orthopedic surgery following a motor vehicular accident after colliding with a tree as an unrestrained driver. He underwent an incision and drainage and intramedullary nail procedure for an open right femur fracture, as well as an incision and drainage and closure of a traumatic left knee arthrotomy at the time. He was also found to have multiple other injuries including a left pubic rami fracture, left knee soft tissue laceration, and multiple bilateral rib fractures. His hospital course during this previous admission was complicated by acute behavioral changes and word-finding difficulties, which raised concerns for a possible stroke. Neurology was consulted where magnetic resonance imaging (MRI) of the brain, as well as computed tomography (CT) scan and magnetic resonance angiography (MRA) of the head and neck, were found to be negative. The electroencephalogram (EEG) was unremarkable. The patient gradually returned to baseline with no intervention, where he was then discharged and transitioned to rehab.

In the emergency department (ED), the patient met two of four Systemic Inflammatory Response Syndrome (SIRS) criteria with a fever of 102 degrees Fahrenheit and tachycardia of 114. His physical examination was significant for swelling and effusion present in his left knee, with a decreased range of motion and tenderness of palpation. X-ray imaging of the left knee was performed and showed degenerative changes in the medial and patellofemoral compartments with no evidence of fracture or joint effusion. Laboratory admission results were significant for elevated C-reactive protein (CRP) and erythrocyte sedimentation rate (ESR) inflammatory markers (Table 1). For this particular admission, the time interval between the laboratory work-up in the emergency department and the onset of AMS was approximately two hours.

**Table 1:** Laboratory results upon admission and after the onset of AMS. Notably, the C-reactive protein levels increased significantly, indicating an inflammatory response.

Components	Results in the emergency department	Results after the onset of altered mental status	Reference
Sodium	134 mmol/L	132 mmol/L	136-145 mmol/L
Potassium	4.4 mmol/L	4.1 mmol/L	3.5-5.1 mmol/L
Chloride	99 mmol/L	99 mmol/L	98-107 mmol/L
Anion gap	9 mmol/L	7 mmol/L	5-12 mmol/L
Blood urea nitrogen	13 mg/dL	12 mg/dL	7-25 mg/dL
Creatinine	0.71 mg/dL	0.6 mg/dL	0.6-1.3 mg/dL
Magnesium	1.6 mg/dL	1.7 mg/dL	1.9-2.7 mg/dL
C-reactive protein	10.02 mg/dL	24.57 mg/dL	<1.00 mg/dL
Erythrocyte sedimentation rate	34 mm/h	-	0-20 mm/h
Lactic acid	0.9 mmol/L	-	0.6-1.4 mmol/L
White blood cell	6.6 x 10^3^/uL	6.9 x 10^3^/uL	4.8-10.8 x 10^3^/uL
Red blood cell	4.45 x 10^6^/uL	3.31 x 10^6^/uL	4.5-6.1 x 10^6^/uL
Hemoglobin	13.9 g/dL	10.1 g/dL	14.0-17.5 g/dL
Hematocrit	41.5%	30.7%	39.0-53.0%
Platelets	211 x 10^3^/uL	233 x 10^3^/uL	130-400 x 10^3^/uL

He was started on intravenous (IV) vancomycin and piperacillin-tazobactam as broad-spectrum antibiotics. Arthrocentesis was performed in the ED, where the synovial fluid had elevated levels of segmented cells, and the culture was only positive for methicillin-resistant *Staphylococcus aureus *(MRSA) (Table 2). He was admitted due to concerns about septic arthritis and was placed on sepsis protocol. He underwent an incision and drainage procedure on his left knee by orthopedic surgery the following morning. Synovial fluid of the left knee was collected during the operation, and cultures were positive for MRSA, methicillin-sensitive *Staphylococcus aureus *(MSSA), and *Pasteurella*. Infectious disease was consulted for antibiotic management, where he was placed on IV vancomycin for MSSA and MRSA, and IV ceftriaxone for *Pasteurella *for four weeks based on culture sensitivity results. The patient's hypomagnesemia was corrected promptly, yet the AMS persisted, suggesting that other factors were contributing to his condition. The elevated CRP levels and positive synovial fluid cultures confirmed an ongoing infection, necessitating a change in antibiotic therapy. This comprehensive analysis underscores the importance of considering multiple etiologies in cases of postoperative AMS.

**Table 2:** Left knee synovial fluid analysis collected by arthrocentesis in the emergency department. The elevated levels of segmented cells in the synovial fluid analysis strongly suggest an infectious process, most likely septic arthritis. This finding supported the clinical presentation and guided the appropriate antibiotic therapy.

Component	Results	Reference
Segmented cells	93%	0-50%
Lymphocytes	1%	0-50%
Monocytes	6%	0-50%
Protein	4.4 g/dL	1.0-3.0 g/dL

Following surgery, the patient developed symptoms of AMS, including speech difficulties, hallucinations, and paranoid behaviors. A comprehensive diagnostic workup ruled out common causes, and psychiatric evaluation revealed severe psychotic and catatonic features. Due to the acute onset of behavioral changes, a CT head without contrast was ordered and found to be stable with no acute intracranial abnormalities unchanged from the previous. Urinalysis was performed and showed no evidence of urinary tract infection given negative leukocyte esterase and nitrites. The urine drug screen was only positive for opioids, which he had been prescribed for pain management since his last admission. Vitamin B12 and thyroid-stimulating hormone (TSH) levels were within normal limits, and Lyme disease and syphilis screenings were negative (Table 3). 

**Table 3:** Further laboratory workup performed for the altered mental status

Components	Results	Reference
Vitamin B12	315 pg/mL	180-914 pg/mL
Lyme disease	<0.90 index	<0.90 index
Treponema pallidum antibody	Non-reactive	Non-reactive
Thyroid-stimulating hormone	1.165 uIU/mL	0.450-5.330 uIU/mL

Psychiatry was consulted, and on examination, the patient was increasingly withdrawn and paranoid, which was similar to his presentation during the previous admission. He was a poor historian at this time and was selectively mute, rarely responding to any questions from the physician. He also displayed acute behavioral changes resembling catatonia, where he was immobile with minimal verbal response. He was able to verbalize orientation with a paucity of speech during attempts to ask other questions. The family members present at the bedside noted that the patient has been under increased stress from working several hours a day, seven days a week for the past five months. During this time, he visited a psychiatrist about three months prior where he was diagnosed with depression and anxiety and was prescribed escitalopram. 

Based on his examination, psychiatry determined that he had a major depressive disorder with sudden onset of recurrent severe psychotic and catatonic features. The patient was given 1 mg of intramuscular lorazepam and 10 mg of ziprasidone for two days to help resolve catatonic symptoms, while his escitalopram was discontinued for possible interactions. The patient gradually improved and returned to baseline after being given lorazepam and ziprasidone. He was then discharged home with antibiotics and prescribed oral ziprasidone 20 mg twice a day, venlafaxine 37.5 mg once a day, and lorazepam 0.5 mg twice a day.

## Discussion

Presented is a case of AMS with catatonic and psychotic features on two separate occasions, with varied degrees of severity and interventions required to return to the patient's baseline state. Such a complex patient history and varied interventions leading to the patient's return to their normal mental status makes the job of identifying the precise etiology and treatments required to alleviate this patient's AMS a challenging one. For such cases, a broad net of differential diagnoses was cast by the physicians with various degrees of success and the need to rule out these diagnoses led to this patient's treatment and eventual return to baseline.

Examining the possible causes of postoperative delirium is necessary to investigate the etiology as there are multiple articles on the subject that can offer insight into some known risk factors that can cause AMS. One study found that the prevalence of non-cardiac surgery-associated delirium can be anywhere between 15% and 54% and can occur as rapidly as 10 minutes post-anesthesia up to seven days [4]. The reported patient presented with several preoperative risk factors including male sex, history of depression, and anxiety. In addition, the use of general anesthesia has been shown to affect chemical balance in the sleep and arousal pathway with the incidence of delirium being lower in patients exposed to light sedation compared to deep sedation [8,9].

The patient's return to the hospital for septic arthritis of his left knee and subsequent AMS with increased severity and catatonic behavior opens the door to broader differentials that all require investigation. It was initially suspected that these acute changes may be from an electrolyte or metabolic etiology requiring rapid blood tests whose values can be evaluated and adjusted relatively quickly. Abnormal levels of serum sodium may present as a profound alteration in mental status or be asymptomatic. However, it is among the most common electrolyte abnormalities encountered in clinical medicine and deserves special attention due to the special care and time required to correct the imbalance safely [10,11]. Articles dating back to 1986 have stressed the importance of calcium homeostasis and the need to investigate its levels in patients with AMS, seizures, and neuromuscular irritability [12]. If abnormalities are found, other complex disorders such as parathyroid dysfunction or malignancy-associated hypercalcemia that may explain these alterations should also be investigated. It should be noted that both hypocalcemia and hypercalcemia can clinically present as symptoms of AMS. Hypokalemia may also serve as a clinical condition to manage in the setting of acute psychosis as it may display a wide range of clinical symptoms including weakness, lethargy, apathy, fatigue, depressed mood, headache, irritability, nervousness, paresthesia, visual disturbances, and muscle discomfort [13]. Lastly, glucose levels should also be carefully investigated as hypoglycemia and hyperglycemia can cause AMS. Hypoglycemia causes symptoms such as confusion, irritability, seizures, and even loss of consciousness as the brain becomes deprived of its primary energy source [14,15]. On the other hand, hyperglycemia in conditions, such as diabetic ketoacidosis (DKA) and hyperosmolar hyperglycemic state (HHS), can lead to AMS due to dehydration, electrolyte imbalances, and the buildup of toxic metabolites. Symptoms of hyperglycemia may include confusion, lethargy, and coma [16]. 

Aside from the metabolic and electrolyte etiologies of AMS, important complications such as infection should also be evaluated [4]. In this patient diagnosed with septic arthritis, there are concerns about the development of sepsis if not immediately treated, which can lead to sepsis-associated encephalopathy (SAE). This condition is characterized by AMS, delirium, and cognitive dysfunction, resulting from the systemic inflammatory response to infection. SAE is a serious complication that requires urgent attention, as widespread inflammation can cause neuroinflammation and disrupt the blood-brain barrier, leading to brain dysfunction. Early recognition of sepsis and the prompt initiation of antibiotics is crucial in managing the infection and preventing the progression of encephalopathy. 

Chemical agents prescribed or administered intraoperatively and postoperatively have been shown to have an impact on a patient's mental status [4]. The patient's postoperative behavioral changes and word-finding difficulties highlight the impact general anesthesia may have on the mental status. Studies have found a propensity to develop mental status changes when patients are sedated with general anesthesia versus spinal anesthesia. Fewer AMS events occurred in the 13,730 patients undergoing total hip arthroplasties who were administered spinal anesthesia than those given general anesthesia (odds ratio, 0.7; p = 0.02) [16]. Postoperative analgesics have a long-documented history of causing AMS with overdone, prolonged use, or in a mixture with other depressive agents such as alcohol. Furthermore, opioid-induced sedation can lead to AMS [16]. 

Perhaps, an interesting and relevant factor in this patient's case is the history of anxiety and his inability to provide a concrete patient history. Benzodiazepines are frequently prescribed medications for the management of anxiety, and we considered the possibility of benzodiazepine withdrawal even without a urine toxicology screen. A case report in 2018 highlighted a 50-year-old male patient with multiple psychiatric comorbidities such as major depressive disorder and generalized anxiety who presented to the ED with AMS, immobility, and mutism. He was initially diagnosed to have had a cerebral vascular accident. However, brain imaging and laboratory tests for that diagnosis were negative. The family of the patient informed his physicians that the patient had stopped taking his 1 mg four times daily alprazolam prescription just four days prior to this patient's catatonic episode that landed him in the ED [17]. The patient's mental status returned to baseline after being given lorazepam 1 mg three times daily. Similarly, another report in 2017 presented a nine-year-old patient with benzodiazepine withdrawal who exhibited similar catatonic behavior [18]. 

Based on these previous studies, it can be stated that postoperative cases of AMS can stem from several etiologies and clinical suspicion bears weight on all aspects of the patient's social history, surgical history, pharmaceutical adherence and interactions, and clinical presentation. Distinguishing between these etiologies can be quite challenging, particularly for critically ill patients with multiple comorbidities. Thus, tailoring the appropriate approach is pivotal in identifying the primary cause of AMS and taking the right steps to correct the offending factor. Fluid and electrolyte disturbances are one of the simplest to identify and correct. They play a significant part in a patient's mental status. Suspicions of life-threatening diagnoses such as sepsis or cerebrovascular accidents require prompt investigation and work-up as delays in treatment can lead to poor clinical outcomes. The timely and tailored approach toward the varied clinical presentation of the presented patient would not have been possible without guidance from other departments including neurology, infectious disease, radiology, and psychiatry. Given the complexity of diagnosing AMS, clinicians should adopt a systematic approach like prioritizing rapid correction of electrolyte imbalances and considering the potential impact of previous psychiatric conditions and medications. Multidisciplinary collaboration is pivotal to investigate and rule out presumed causes of AMS. This will ultimately help narrow down treatment options to best fit this patient's clinical presentation and lead to the alleviation of their symptoms and return to baseline. 

## Conclusions

Postoperative AMS presents a complex diagnostic challenge that requires a thorough and systematic approach. This case report underscores the importance of considering a broad differential diagnosis when evaluating postoperative AMS, including central nervous system causes, metabolic disturbances, drug-related factors, and systemic conditions. Early recognition and intervention, including rapid correction of electrolyte imbalances and close monitoring of psychiatric symptoms, are crucial in managing postoperative AMS. Optimizing patient outcomes and minimizing complications in the postoperative setting, especially for those at higher risk due to older age, preexisting cognitive impairment, or significant comorbidities, requires close monitoring, and preventative strategies. Interdisciplinary collaboration is essential for optimizing patient outcomes. Future research should focus on developing standardized protocols for the diagnosis and management of postoperative AMS.
